# Arrival Time Correction for Dynamic Susceptibility Contrast MR Permeability Imaging in Stroke Patients

**DOI:** 10.1371/journal.pone.0052656

**Published:** 2012-12-20

**Authors:** Richard Leigh, Shyian S. Jen, Daniel D. Varma, Argye E. Hillis, Peter B. Barker

**Affiliations:** 1 Department of Neurology, Johns Hopkins University School of Medicine, Baltimore, Maryland, United States of America; 2 Flinders Comprehensive Stroke Centre, Bedford Park, Australia; 3 Russell H. Morgan Department of Radiology and Radiological Science, Johns Hopkins University School of Medicine, Baltimore, Maryland, United States of America; 4 F.M. Kirby Research Center for Functional Brain Imaging, Kennedy Krieger Institute, Baltimore, Maryland, United States of America; University of Manchester, United Kingdom

## Abstract

**Purpose:**

To determine if applying an arrival time correction (ATC) to dynamic susceptibility contrast (DSC) based permeability imaging will improve its ability to identify contrast leakage in stroke patients for whom the shape of the measured curve may be very different due to hypoperfusion.

**Materials and Methods:**

A technique described in brain tumor patients was adapted to incorporate a correction for delayed contrast delivery due to perfusion deficits. This technique was applied to the MRIs of 9 stroke patients known to have blood-brain barrier (BBB) disruption on T1 post contrast imaging. Regions of BBB damage were compared with normal tissue from the contralateral hemisphere. Receiver operating characteristic (ROC) analysis was performed to compare the detection of BBB damage before and after ATC.

**Results:**

ATC improved the area under the curve (AUC) of the ROC from 0.53 to 0.70. The sensitivity improved from 0.51 to 0.67 and the specificity improved from 0.57 to 0.66. Visual inspection of the ROC curve revealed that the performance of the uncorrected analysis was worse than random guess at some thresholds.

**Conclusions:**

The ability of DSC permeability imaging to identify contrast enhancing tissue in stroke patients improved considerably when an ATC was applied. Using DSC permeability imaging in stroke patients without an ATC may lead to false identification of BBB disruption.

## Introduction

Magnetic resonance imaging (MRI) of the blood-brain barrier (BBB) can be achieved using dynamic contrast enhanced (DCE) T1-weighted imaging. This methodology has been well described [1] and involves calculation of a measure of permeability, K^trans^. Although DCE MRI has been shown to be a robust research tool, it has yet to become part of standard clinical practice. In part this is due to the time-consuming process of acquiring the images required for generating permeability measures with DCE MRI.

Conversely, dynamic susceptibility contrast (DSC) MRI is a routinely acquired imaging technique most commonly used in ischemic stroke patients or brain tumor patients. In brain tumor patients DSC MRI is used to measure cerebral blood volume (CBV) of the tumor as this has been associated with tumor grade [2]. However, leakage of contrast due to BBB disruption can lead to an underestimation of CBV [3]. A method for contrast leakage has been described [4] and applied to brain tumor patients [3]. In order to correct for BBB disruption, a measure of permeability is extracted from the DSC MRI acquisition. This approach generates a measure that has been labeled K_2_ which is an estimate of K^trans^. DSC MRI is routinely collected on acute stroke patients at many large academic medical centers as part of the evaluation for treatment [5]. In this setting it is referred to as perfusion weighted imaging (PWI) and provides information about the blood flow to the brain. Several groups have attempted to extract permeability information from PWI in stroke [6]–[8]. However, the approach used in the brain tumor literature, which assumes symmetric perfusion of the brain, can be subject to error when applied to patients with perfusion deficits, such as acute stroke patients. The delay in contrast delivery to areas of hypoperfusion makes calculation of K_2_ inaccurate. Thus we developed a technique that applies an arrival time correction (ATC) prior to calculation of K_2_. The purpose of this study was to compare K_2_ measurements made with and without ATC in a population of stroke patients who were known to have damage to the BBB based on T1-post contrast imaging.

## Materials and Methods

### DSC MR Permeability Imaging

There are several approaches to using DSC MRI to assess the permeability of the BBB that have been described in the literature [3], [4], [6]–[8]. However, none of these techniques perform an ATC. We chose the method described by Boxerman et al. to use as the uncorrected method of DSC MRI permeability imaging. This technique has been described in detail elsewhere [3] but will be briefly summarized here.

Changes in tissue contrast agent concentration are measured as changes in relaxivity with the equation [4]:
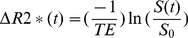
(1)


Where 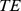 is the time to echo, 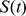 is the signal intensity in the voxel at time 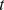, and 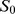 is the baseline signal intensity prior to delivery of the contrast bolus. When contrast leaks through the BBB into the parenchyma the measured signal is more accurately characterized by adding a term to equation (1) to account for 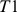 effects [4]:
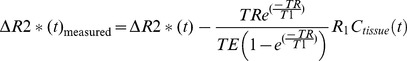
(2)


Where 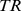 is the time to repetition, 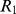 is 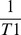, and 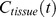
is the concentration of contrast in the tissue at time 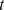. The amount of contrast leakage for each voxel is estimated by assuming that the measured relaxivity change is a linear combination of the average signal in non-enhancing voxels and some fraction of its time integral:
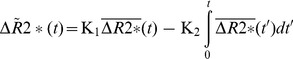
(3)


Where 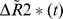 is the measured, uncorrected change in relaxivity, 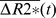 is the average signal for a region of non-enhancing voxels, and 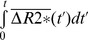 is the integral of the average signal for a region of non-enhancing voxels which is essentially the average cerebral blood volume (CBV). The term K_1
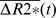
_ represents the uncontaminated portion of the measured signal as the average signal of non-enhancing values times a scaling factor K_1_. The K_2
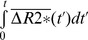
_ term reflects the effect due to leakage and is represented as the average CBV of non-enhancing tissue times K_2_ where K_2_ is a fraction between 0 and 1. Thus when equation 3 is solved for K_2_, the fraction of the average CBV that has leaked at each voxel is approximated.

### Arrival Time Correction

Inherent in the MRI DSC permeability imaging technique described above is the assumption that the recorded signal for a given voxel can be represented as a scaled version of the average signal. For example, if a voxel had no contrast leakage, K_2_ would be zero and equation 3 would become:
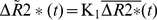
(4)


However, this assumption fails when there is a delay in contrast delivery such as in a perfusion deficit of a stroke patient. The shape of the measured curve is often very different in hypoperfused tissues. As the curve becomes broader, it peaks later and has a different area underneath it. Thus our approach to performing an ATC was to adjust 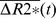 on a voxel by voxel basis so that it best fits the true morphology of the recorded signal. We defined a term:
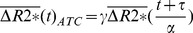
(5)


**Figure 1:**
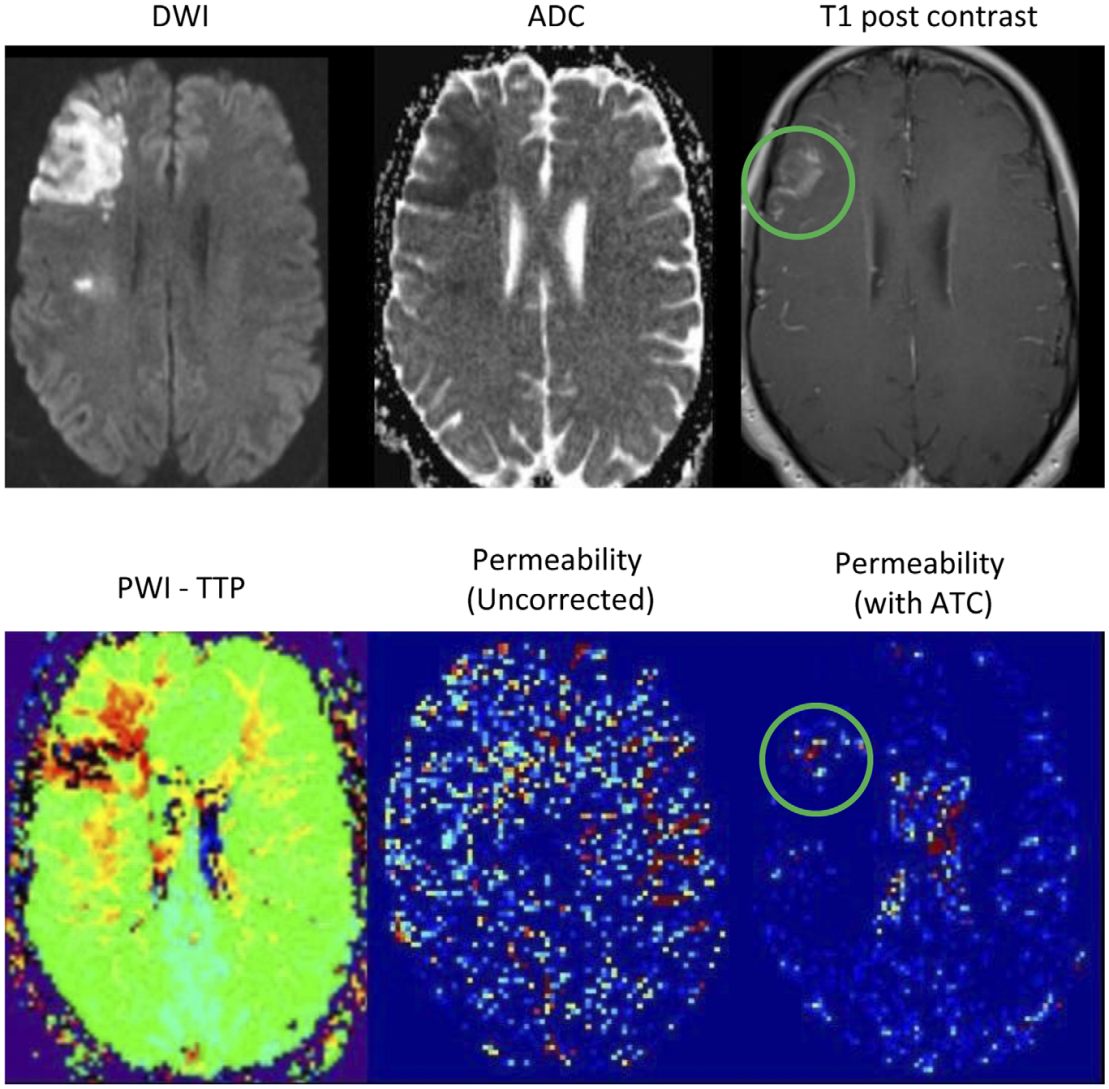
The top row of images show a stroke on DWI/ADC which has some enhancement on post contrast T1 imaging. The bottom row shows the perfusion deficit on a TTP map, the permeability image when not corrected for arrival time, and the permeability image after arrival time correction. The green circles show corresponding areas of contrast leakage on the T1 post contrast and ATC permeability images. (DWI = diffusion weighted image, ADC = apparent diffusion coefficient, PWI = perfusion weighted image, TTP = time to peak, ATC = arrival time corrected).

**Figure 2:**
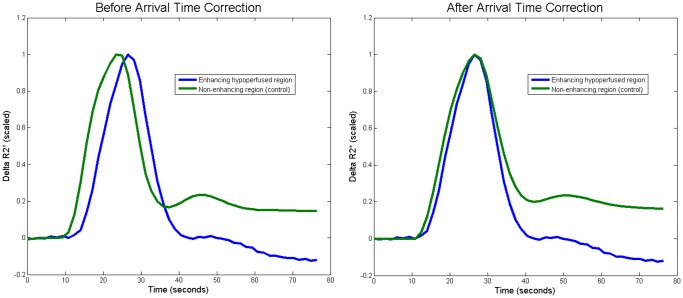
The two graphs show the ΔR2* for non-enhancing (control) and enhancing hypoperfused regions before and after arrival time correction (ATC). In the first graph, due to even a small delay in time-to-peak, the control signal appears to approach baseline faster thus obscuring the phenomenon being measured. However, in the second graph, after the ATC has been applied to the control, it becomes evident that the enhancing region signal is approaching the baseline faster due to the T1 effect of contrast accumulation in the parenchyma.

Where 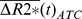 is the average signal after ATC, 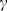 is a magnitude scaling factor, 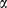 is a time scaling factor, and 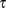 is a time offset. Thus equation 3 becomes:
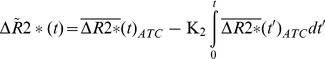
(6)where K1 is dropped because scaling has been performed as part of the ATC. Using a multiple least-squares approach, the values for 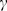, 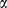 and 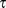 are determined by minimizing the following equation over a range of values:



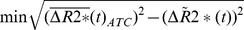
(7)Thus, at every voxel 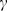, 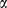 and 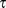 are determined to create an ATC non-enhancing curve to compare with the recorded signal to determine if there is evidence of BBB disruption. The K_2_ value generated again represents the fraction of the CBV that has leaked. However, in this case, the K_2_ value is the fraction of the CBV calculated using the corrected concentration curve. An example of the K_2_ permeability images before and after ATC is shown in Figure 1. An example of how the non-enhancing curve is adjusted with the ATC is shown in Figure 2.

### Patient Selection

This study was approved by the Johns Hopkins Institutional Review Board and did not require consent. Consent was not required by our IRB for this de-identified database due to the retrospective nature of the study and the lack of patient interaction. MRIs of all patients admitted to our stroke service over the time period from 8/1/2010–3/31/2011 were retrospectively reviewed by one author to identify evidence of BBB disruption characterized by enhancement on T1 post-contrast (T1PC) images. Two other authors blinded to whether patients had been identified as having BBB disruption reviewed a collection of patients with and without BBB disruption and independently determined if they had evidence of enhancement on T1 post-contrast (T1PC) images. There was agreement between all three authors on all patients included in the study except for one patient for whom only two reviewers identified BBB damage. However, since the official radiology report for the scan in question commented on the presence of enhancement on T1 post-contrast (T1PC) images, this patient was included in the study. Patients with BBB disruption, a successful DSC MRI scan, and an acute stroke on diffusion weighted imaging (DWI) were included in the analysis. If a patient had more than one MRI done during their hospital stay that met the inclusion criteria, then all eligible scans were included. Posterior circulation strokes were excluded due to potential artifacts in perfusion imaging at the base of the skull. Patients with evidence of hemorrhagic transformation on perfusion source images were excluded due to the artifact caused by hemosiderin deposition on gradient-echo echo-planar images.

### Image Acquisition

Images were acquired as part of routine clinical care; thus there was no standardization of imaging parameters. Our institution has several MRI scanners with magnet strengths ranging from 1.5–3 Tesla. Siemens, Philips and General Electric brand scanners were used. Although the protocols varied, all patients included in the analysis had serial echoplanar gradient-echo images acquired prior to and during the injection of a weight-based dose of gadopentetate dimeglumine (Magnevist; Bayer HealthCare Pharmaceuticals). T1 weighted imaging was performed after the perfusion acquisition.

### Image Processing

All image analysis was done with Matlab (Mathworks, Natick, MA, USA). K_2_ images were generated from DSC images as described above. Using T1 post contrast images as a guide, regions of interest (ROI) were outlined in the ischemic hemisphere to delineate BBB disruption on the DSC source images. This was done visually by bringing up the T1 post contrast images next to the T2* baseline DSC source images. The ROI was then flipped into the contralateral hemisphere to create a control ROI.

### Receiver Operating Characteristic (ROC) Analysis

Voxels in the ROI of the ischemic hemisphere were designated as having BBB disruption, while voxels from the control ROI were designated as no BBB disruption. K_2_ values are calculated in the permeability analysis as a fraction of the CBV, thus they range from 0 to 1. Using the K_2_ values from the permeability analysis, voxels can be divided into 2 groups based on a threshold which is varied from 0 to 1. For instance, a threshold of 0.2 would indicate that 20% of the CBV would have to be measured as leakage on the K_2_ image in order for it to be considered as representative of true BBB disruption. For every given threshold the classification of voxels as having BBB damage or not will result in true positives, false positives, true negatives and false negatives. Thus for every threshold a sensitivity and specificity is generated for both the corrected and uncorrected permeability images. Plotting sensitivity versus 1-specificity results in an ROC curve. A perfect test would result in a curve that intersects the top left hand corner. This would indicate that a threshold was identified that has a sensitivity of 1 and a specificity of 1. The ROC curve for a random guess results in a diagonal line from bottom left to top right. The area under the curve (AUC) is a measure of the overall performance of the test. For a perfect test the AUC would be 1 while the AUC for a random guess curve would be 0.5. ROC curves were generated to compare the performance of the corrected versus uncorrected images at identifying BBB damage.

## Results

Nine patients and 13 MRI scans met the inclusion criteria and were included in the analysis. The mean age of the patients was 60 years old, and 6 of the patients were female. The time from stroke to MRI scan ranged from 2 hours and 44 minutes to 10 days, 7 hours and 41 minutes. The median time from stroke to MRI was 2 days, 1 hour and 52 minutes. Four of the 9 patients had a witnessed time of stroke onset. For the remaining patients the “last known normal” time was used. Four patients had left hemisphere anterior circulation strokes, and 5 patients had right hemisphere anterior circulation strokes.

**Figure 3:**
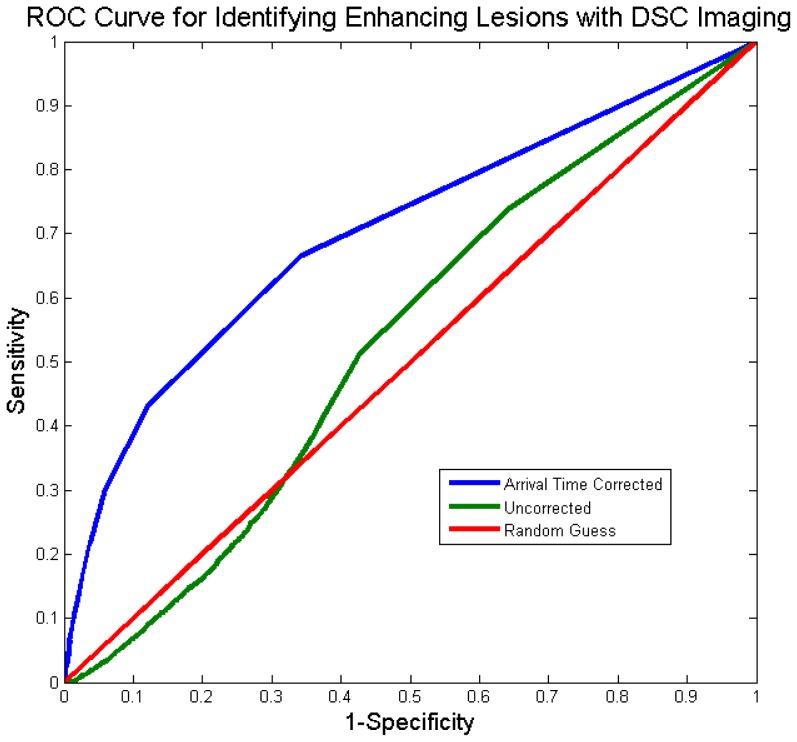
The receiver-operator characteristic (ROC) curves, which demonstrate the ability to correctly identify enhancing tissue, are plotted before and after arrival time correction (ATC). Prior to correction the technique performs worse than random chance at some thresholds due to perfusion deficits.

The ROC curves are plotted for the uncorrected and the ATC techniques in Figure 3. The AUC for the uncorrected method was 0.53. ATC improved the ability to detect BBB disruption to an AUC of 0.70. Thresholds of 0.005 (0.5% of CBV) for the uncorrected and 0.011 (1.1% of CBV) for the corrected were identified as the best (i.e. resulted in a sensitivity/specificity point closest to the top-left hand corner of the ROC curve). The sensitivity improved from 0.51 to 0.67 and the specificity improved from 0.57 to 0.66. Visual inspection of the ROC curve reveals that the performance of the uncorrected analysis was worse than random guess at some thresholds.

## Discussion

We set out to determine whether applying an arrival time correction (ATC) improves the ability of perfusion-weighted imaging to detect breakdown of blood-brain barrier following stroke. Our study confirms that DSC permeability imaging is improved using this correction. How does our finding impact on current approaches to imaging in acute stroke patients?

The role of BBB disruption in stroke patients has been investigated by a variety of measurement techniques [6], [7], [9]–[14]. These studies are driven by the idea that damage to the BBB in acute stroke patients may predict response to treatment. More specifically, damage to the BBB may provide a measure of the risk of intracranial hemorrhage (ICH), which is the most serious complication of thrombolytic stroke treatment. T1 post contrast imaging is the most commonly used clinical method for detecting damage to the BBB. It has been shown to be very specific for predicting ICH in stroke patients but not very sensitive [12]. FLAIR post contrast imaging, referred to as hyperintense acute reperfusion marker (HARM), has also been investigated and has been shown to predict hemorrhagic transformation and poor outcome in stroke patients [14]. However, this approach requires a delay between the administration of contrast and image acquisition on the order of hours and thus is not practical for management of acute stroke, which takes place on the order of minutes. The use of DSC MRI to detect BBB has also been investigated using various approaches [6]–[8], but none of these approaches uses an ATC prior to calculating permeability. Despite this body of literature, permeability imaging has not found its way into clinical use [5].

Our study does not investigate the role of BBB in stroke patients. It was not designed to validate the use of DSC MRI to detect damage to the BBB. Furthermore, it does not provide any information about how DSC MRI permeability measures might be used in acute stroke patients. These are all important questions that will need to be addressed in future studies.

This study was formulated to answer a very specific question, “In stroke patients with evidence of BBB disruption, does ATC of DSC MRI improve identification of permeability derangements?” Based on the ROC analysis of this study, ATC improves the performance of DSC MRI based permeability imaging as defined by T1 post contrast imaging. The ROC analysis also reveals that in the absence of ATC, DSC MR based permeability imaging can be worse than random guess at identifying damage to the BBB. This is not unexpected, since perfusion deficits, when not corrected for, can be erroneously identified as permeability derangements due to assumptions of the model. Specifically, the model assumes that the shape of the curve of the recorded signal will be the same throughout the brain, even in hypoperfused tissue. In reality, the recorded curve in hypoperfused tissue has a different morphology. Thus, ATC of the recorded curve adjusts its morphology and improves the performance of the model.

This study has several limitations. The use of T1 post contrast imaging as a gold standard is not ideal. Furthermore the user defined ROIs are somewhat subjective. Additionally, the lack of a standard DSC protocol and the use of multiple scanner types and strengths add a degree of variability that is not controlled for. The signal detected with K_2_ permeability imaging, even after ATC, is modest and can be difficult to differentiate from noise. However, much of the noise signal is found in the blood vessels and the ventricular system, not in the brain parenchyma. For this study we explicitly identified ROIs within brain parenchyma, thus application of this technique requires knowledge of the underlying structures.

Despite its limitations, this study serves to describe a technique for ATC of DSC MRI permeability imaging which can be further tested in subsequent studies.
